# Circular RNAs in neurological conditions – computational identification, functional validation, and potential clinical applications

**DOI:** 10.1038/s41380-025-02925-1

**Published:** 2025-02-17

**Authors:** Oak Hatzimanolis, Alex M. Sykes, Alexandre S. Cristino

**Affiliations:** Institute for Biomedicine and Glycomics, Griffith University, Brisbane, QLD Australia

**Keywords:** Molecular biology, Psychiatric disorders

## Abstract

Non-coding RNAs (ncRNAs) have gained significant attention in recent years due to advancements in biotechnology, particularly high-throughput total RNA sequencing. These developments have led to new understandings of non-coding biology, revealing that approximately 80% of non-coding regions in the genome possesses biochemical functionality. Among ncRNAs, circular RNAs (circRNAs), first identified in 1976, have emerged as a prominent research field. CircRNAs are abundant in most human cell types, evolutionary conserved, highly stable, and formed by back-splicing events which generate covalently closed ends. Notably, circRNAs exhibit high expression levels in neural tissue and perform diverse biochemical functions, including acting as molecular sponges for microRNAs, interacting with RNA-binding proteins to regulate their availability and activity, modulating transcription and splicing, and even translating into functional peptides in some cases. Recent advancements in computational and experimental methods have enhanced our ability to identify and validate circRNAs, providing valuable insights into their biological roles. This review focuses on recent developments in circRNA research as they related to neuropsychiatric and neurodegenerative conditions. We also explore their potential applications in clinical diagnostics, therapeutics, and future research directions. CircRNAs remain a relatively underexplored area of non-coding biology, particularly in the context of neurological disorders. However, emerging evidence supports their role as critical players in the etiology and molecular mechanisms of conditions such as schizophrenia, bipolar disorder, major depressive disorder, Alzheimer’s disease, and Parkinson’s disease. These findings suggest that circRNAs may provide a novel framework contributing to the molecular dysfunctions underpinning these complex neurological conditions.

## Introduction

Unravelling the intricate genetic mechanisms underlying non-coding RNA (ncRNA) biology has emerged as a promising frontier for the development of new therapeutic and diagnostic tools for neurological conditions. ncRNAs have gained significant attention as evidence accumulates regarding their biochemical functionality, which was once underestimated [1–5]. Approximately 80% of the human non-coding transcriptome is now predicted to be biochemically active [1, 3, 6, 7]. A notable class of ncRNA are circular RNAs (circRNAs) [8–14]. CircRNAs are highly stable, covalently closed-looped, non-linear RNAs generated by circularizing back splicing events. Most circRNAs are derived from exonic regions of protein-coding genes [8, 13]. Multiple circRNA isoforms can arise from a single gene, depending on splicing processes involved, with circRNAs transcribed from combinations of exons, introns, intergenic, and untranslated regions [8, 13, 15]. These transcripts are often co-transcriptionally generated with pre-mRNA on the same strand and are known to compete with mRNA for expression levels [9, 16]. While the majority of circRNAs are derived from the nuclear genome, a subset has been identified originating from the mitochondrial chromosome [17, 18].

Since circRNAs were first identified in 1976, they were largely dismissed as “junk RNA”, with only a few being recognized as biochemically functional, primarily in the context of viral biology [19–21]. However, over the past decade, circRNAs have become an area of high-impact research, with many circRNA transcripts identified across species, from archaea to humans [22–24]. Many circRNAs have high sequence conservation across different species, and over 150,000 unique human circRNAs have been computationally identified, with a subset experimentally validated. CircRNAs are expressed throughout the human body, displaying cell-type and tissue-specific expression patterns. For example, distinct profiles have been observed in neuronal cells such as dopaminergic neurons, pyramidal neurons, and glia [25–27]. Furthermore, circRNAs are predominantly expressed in the mammalian brain at levels significantly higher than those in other tissues [26, 28, 29]. The temporal expression of circRNAs appears to be dynamic during early neuronal development and shows increased levels with age [30–32]. Dysregulated circRNA expression has been reported across various neurological conditions, often linked to altered physiological processes and functional differences [27, 33–37]. In this review, we focus on five conditions – schizophrenia, bipolar disorder, major depressive disorder, Alzheimer’s disease, and Parkinson’s disease – highlighting key findings from human and rodent studies to explore recent discoveries in circRNA biology and their relevance to neurological disorders.

New advancements in molecular biology and computer science have shed light on what was once considered a “junk” portion of the genome. A wide range of ncRNAs has now been shown to possess biochemical functionality, playing critical roles in cellular processes such as transcriptional regulation [38, 39], post-transcript processing [10, 14], protein expression [40–42], and splicing regulation [9, 43]. While the full extent of their impact on brain activity remains to be elucidated, ncRNAs, including circRNAs, are proving to be invaluable tools for understanding the etiology and pathophysiology of neurological conditions, as well as potential candidates for clinical applications.

## The molecular properties of circRNAs

The precise mechanisms of the biogenesis, transport, localization and degradation of circRNAs remain largely unresolved. However, current research indicates that human circRNAs are predominantly composed of exonic regions (~60%), with most circRNAs consisting entirely of protein-coding exons. Exonic circRNAs originate from pre-mRNAs that first undergo canonical splicing followed by backsplicing, and most are generated co-transcriptionally in a spliceosome-dependent manner [44, 45]. Typically, these circRNAs consist of two or three exons, with intervening introns excised during the splicing process [46]. In addition to exonic circRNAs, they can also arise from other genomic regions, including intronic-exonic, fully intronic, and intergenic regions [47–49]. Beyond biogenesis, circRNA functions are influenced by several molecular properties, including subcellular transport and localization, degradation pathways, and protein translation capacity, which are discussed in the following sections.

### Biogenesis

**Fig. 1:**
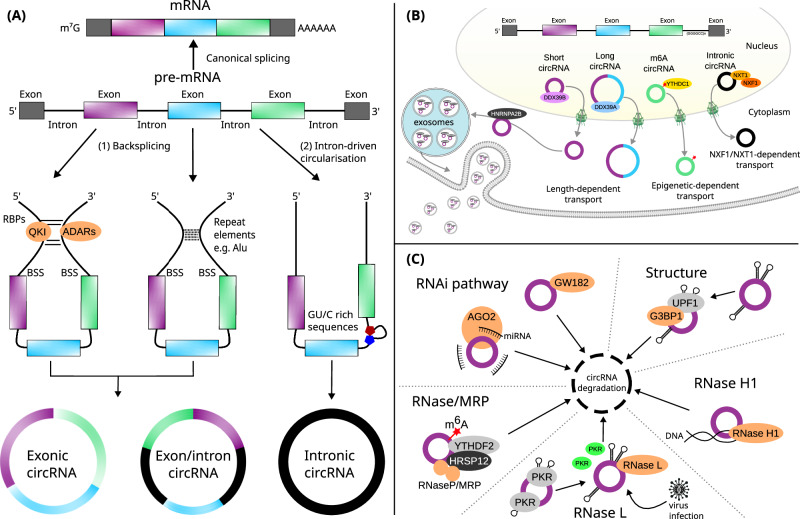
CircRNA biogenesis, molecular properties, and degradation pathways. (**A**) CircRNA biogenesis is predominantly explained by two proposed models: (1) direct backsplicing, facilitated by binding sites recognized by RNA binding proteins (RBPs) or inverted repeat elements that bring splice sites into close proximity; and (2) the lariat-driven circularization model, where exon skipping generates a lariat structure that is subsequently processed into an intron-driven circularization. BSS backsplicing sites. (**B**) The nuclear export of circRNAs is dependent on their length, methylation status, sequence origin, and interactions with nuclear proteins. Transport into extracellular spaces is mediated by selective exosomal transport, which may operate through mechanisms unique to circRNAs, distinct from those of other cellular components. (**C**) CircRNA degradation involves several pathways, including those mediated by RNA interference (RNAi), endoribonucleases (RNAses), and structural features of circRNAs.

Several distinct mechanisms contribute to circRNA biogenesis (Fig. 1A). Most circRNAs are generated through a process called backsplicing, where the canonical 5′ and 3′ splice sites of a pre-mRNA are joined in reverse orientation, forming a covalent 3–5′ phosphodiester bond [13, 50]. This process is tightly regulated by trans-acting factors such as RNA binding proteins (RBPs), including QKI (QKI, KH Domain Containing RNA Binding) [51] and NOVA2 (NOVA Alternative Splicing Regulator 2) [52], which dimerize, causing increased proximity of splice sites through specific motifs within the upstream and downstream intronic regions. Cis-acting elements also play critical role in circRNA formation, for example, flanking intronic base-pairing of reverse complementary sequences (e.g. *Alu* elements [12]) located near the splice sites can pair with one another, facilitating circRNA biogenesis through a mechanism similar to the interaction between RBPs.

Adenosine deaminases acting on RNA (ADARs) play an important role in circRNA biogenesis by editing RNA through adenosine to inosine (A-to-I) conversions. This editing is guided by the base-pairing of reverse complementary sequences, which modulates the stability of RNA secondary structures. By altering these structures, ADARs regulate the accessibility of regulatory RBPs, thereby either reducing or enhancing back-splicing events [53, 54]. In addition, the spliceosome machinery is thought to directly participate in circRNA formation, akin to canonical splicing, although the precise mechanisms remain to be fully elucidated [9, 13, 50]. In the intron lariat-driven circularization model, an internal backsplicing event occurs after exon skipping, producing a mRNA that lacks the skipped exons and a circRNA can be formed if the lariat structure escapes debranching. Sequence elements such as a 7-nucleotide GU motif and an 11-nucleotide C-rich sequence near the 5′ splice sites protect the lariat from degradation by debranching enzymes (e.g. Debranching RNA Lariats 1, DBR1), promoting circRNA formation [47, 55]. Moreover, N^6^-methyladenosine (m^6^A) RNA modification, which are known to regulate various aspects of RNA metabolism, including splicing, stability, and translation, have also been implicated in circRNA biogenesis. Specifically, m^6^A-modified exons located near the start and stop codons of mRNAs can undergo backsplicing mediated by the nuclear m^6^A reader protein YTHDC1 (YTH N6-Methyladenosine RNA Binding Protein C1) [56–58].

### Subcellular transport and localization

Since circRNAs are generated in the nucleus and found across various subcellular compartments, with exon-containing circRNAs predominantly localized in the cytoplasm [12, 59, 60], their nuclear export is likely to follow a tightly regulated process. Multiple mechanisms involving RBPs, export receptors, and RNA helicases facilitate circRNA nuclear export (Fig. 1B). The primary mechanism of circRNA nuclear export relies on Ran-GTP binding export receptors [61, 62] which transport circRNAs through the nuclear pore complex. In mammalians, exportin-2 (XPO2) plays a key role, exporting approximately 80% of the most abundantly expressed circRNAs [62]. Another important exportin, exportin-4 (XPO4), is responsible for transporting a distinct subset of exonic circRNAs, which are highly expressed in brain tissues [61].

In addition to Ran-GTP-dependent exportins, length-dependent mechanisms involving RNA helicases have been identified. DDX39A (DExD-Box Helicase 39 A) and DDX39B (DExD-Box Helicase 39 B) are required for the export of short (less than 700nt) and long circRNAs (greater than 800nt), respectively. This length-dependent export mechanism has been observed across metazoans, suggesting it is evolutionarily conserved [63, 64]. Interestingly, canonical mRNA export factors, such as NXF1 (Nuclear Export Factor 1), ALYREF (ALY/REF Export Factor), and GANP (Germinal Center-Associated Nuclear Protein), have minimal involvement in circRNA export [62]. This highlights the distinct nature of circRNA export pathways, which are mechanistically different from those of linear RNAs. Nonetheless, the NXF1-NXT1 pathway, a known mRNA export system, has been implicated in transporting GC-rich intronic circRNAs, demonstrating the diversity and complexity of circRNA export mechanisms [65].

Moreover, RNA methylation, particularly N6-methyladenosine (m^6^A), plays an important role in circRNA export. The nuclear protein YTHDC1, which also participates in the m^6^A-dependent biogenesis of specific circRNA subsets [56–58], mediates the export of m^6^A-modified circRNAs, including circNSUN2 [66] and circRNA3634 [67]. However, not all m^6^A -modified circRNAs rely on YTHDC1 for export. For instance, circ-ZNF609 biogenesis is reduced following YTHDC1 knockdown, but its export and stability remain unaffected [57], indicating the involvement of alternative mechanisms in the export of m^6^A -modified circRNAs.

CircRNAs have also been identified in exosomes [68–73], a class of extracellular vesicles that originate from intraluminal vesicles within multivesicular endosomes. Exosomes play key roles in intercellular communication, often transporting RNAs, proteins and lipids [74, 75]. Interestingly, circRNAs are enriched in exosomes relative to their abundance in the cells of origin, indicating selective transport into exosomes [69]. Although research on exosomal circRNAs is still emerging, most evidence so far comes from studies in cancer and immunology. Several possible mechanisms have been proposed for the selective packaging of circRNAs into exosomes, such as RBP recognition of specific binding sequences [76–78] resembling the selective export of small RNAs [79–81]. Other potential mechanisms involve long non-coding RNAs (lncRNAs) [82], microRNAs (miRNAs) [69], or circRNAs [70] size as determinants of selective transport. However, the exact pathways by which circRNAs are selectively transported into exosomes remains unclear.

Despite the progress made in understanding circRNA transport and subcellular localization, many questions remain to be resolved. The involvement of additional RNA export-related factors is still unclear, and it is likely that future research will identify new pathways or proteins contributing to the transport of specific circRNA subsets. Furthermore, specialized transport mechanism or tissue-specific factors may play essential roles in organs such as the brain, where circRNAs are highly expressed and found in neurites and synaptosomes [29, 83, 84].

### Stability and degradation

The covalently closed-loop structure of circRNAs, which lacks free 5′ and 3′ ends, makes them significantly more stable and resistant to degradation by exonucleases compared to their linear counterparts [85]. In mammalian cells, most circRNAs have a half-life of 18.8–23.7 h, making them stable for at least 2.5 times longer than linear RNAs, which have a half-life of 4.0–7.4 h [86]. This extended half-life suggests that the circRNA decay machinery differs substantially from that of linear RNA, with several molecular properties affecting circRNA stability and degradation, including endonuclease activity mediated by miRNA binding, secondary structure, RNA-DNA duplexes, and m^6^A modification (Fig. 1C).

CircRNAs can be degraded through endonucleases via RNA interference (RNAi) pathways. The Argonaute-2 (AGO2) endonuclease, guided by miRNA, has been implicated in circRNA degradation. For instance, miR-1224 binds to the precursor circRNA-filip1l in the nucleus of mice spinal cord neurons, reducing circRNA-filip1l expression in an AGO2-dependent manner [87]. Another example is miR-671, which directs AGO2-mediated cleavage of the circular transcript from the *long intergenic non-protein coding RNA 632* gene (circLINC00632 also known as circCDR1as) [88], a process that plays an important role in brain function [89]. Additionally, GW182 (Trinucleotide Repeat Containing Adaptor 6 A, TNRC6A), a key component of the RNAi pathway, is involved in the degradation of circRNAs via RNAi machinery-independent pathways [90].

Highly structured circRNAs can be degraded by RBP complexes. For example, the UPF1 RNA helicase unwinds circRNA secondary structures, enabling degradation by the endonuclease G3BP1 (G3BP Stress Granule Assembly Factor 1) [91]. Some circRNAs, such as ci-ankrd52, can form DNA:RNA hybrids at their transcription sites which maintains an open secondary structure forming a stable R-loop with the template DNA that is recognized and degraded by RNase H1 [92]. Moreover, during viral infections, circRNAs forming 16–26 bp RNA duplexes can be cleaved by RNase L, an endonuclease activated in response to viral infection. This degradation is necessary to activate PKR, a double stranded RNA-activated protein kinase, which limits viral and host protein synthesis [93–95]. CircRNAs with m^6^A modifications are targeted for degradation by the RNase-P/MRP complex. This process requires HRSP12 (Reactive Intermediate Imine Deaminase A Homolog, RIDA), which acts as a bridge between the m^6^A reader protein YTHDF2 (YTH N6-Methyladenosine RNA Binding Protein F2) and RNase-P/MRP, facilitating the rapid decay of m^6^A-modified circRNAs [96, 97].

Currently, there is no evidence for a canonical degradation pathway specific to circRNAs. The available research suggests that multiple cellular pathways, some of which are shared with linear RNAs, contribute to circRNA degradation. However, the relative importance of these pathways and whether novel mechanisms exclusive to circRNAs exist, remain to be fully elucidated. Further research is needed to uncover the dominant mechanisms underlying circRNA stability and decay.

## The mechanisms of action of circRNAs

### microRNA sponge

**Fig. 2:**
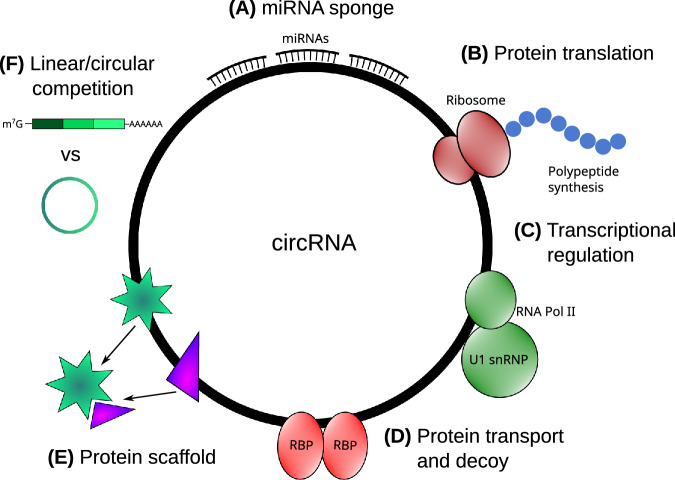
Diagram summarizing mechanisms of actions and functions of circRNAs. (**A**) One of the most extensively studied functions of circRNAs is miRNA sponging, where circRNAs bind to complementary miRNA binding sites, sequestering miRNAs and reducing their inhibitory effect on target mRNAs. (**B**) CircRNAs can encode functional peptides through m^7^G cap-independent translation mechanisms, facilitated by internal ribosome entry sites (IRES) or N6-methyladenosine (m6A) modifications. (**C**) CircRNAs regulate transcription by interacting with transcriptional factors or spliceosome components. (**D**) Many circRNAs contain binding sites for RNA binding proteins (RBPs), regulating the localization, stability, or activity of these proteins. (**E**) CircRNAs can act as scaffolds in protein-protein interactions, binding to multiple proteins simultaneously to facilitate their functional interplay. (**F**) CircRNAs are often co-transcriptionally produced with mRNAs from the same host gene, competing with linear transcripts for splicing events, thereby interfering with the expression of their cognate mRNAs.

MiRNA sponging is a well-known function of circRNA (Fig. 2A), in which circRNAs sequester miRNAs through complementary binding sequences, reducing their bioavailability. This sponging activity inhibits miRNA-mediated gene silencing by preventing miRNAs from binding to their target mRNA transcripts. Typically, miRNAs form complexes with AGO2, and their seed region (a conserved 2–8 nucleotide sequence) binds to the 3′ UTRs of mRNAs, leading to degradation of mRNA transcripts containing complementary miRNA response elements [98, 99].

One of the most prominent examples of miRNA sponging is circCDR1as, which contains over 60 conserved binding sites for miR-7 [100, 101]. This large number of binding sites and high expression relative to most circRNAs, enables circCDR1as to tightly regulate miR-7 availability, potentially affecting the expression of several target genes. Dysregulation of miR-7 has been shown to impair the development and function of the brain and pancreas [102, 103] and is also implicated in Parkinson’s disease through dysregulation of α-synuclein expression [104]. In line with this, abnormal expression of circCDR1as has been found to play important roles in brain development [101], insulin production and secretion [39], as well as in promoting cell proliferation and metastasis in cancers such as nasopharyngeal carcinoma [105], osteosarcoma [106], and melanoma [107].

Several other instances of miRNA sponging have been identified, suggesting that circRNAs may act as miRNA decoys, representing a key regulatory mechanism. For example, the testis-specific circRNA sex-determining region Y (circSry) functions as a sponge for miR-138, though the functional impact remains to be determined [100]. CircNRIP1 acts as a decoy for miR-149, and is transmitted between gastric cancer cells via exosomes, where it affects the AKT1/mTOR pathway to promote tumor metastasis [108]. In bladder cancer, circHIPK3 contains two binding sites for miR-558, where sponging of miR-558 suppresses heparinase (HPSE) expression [109].

As research into circRNA sponging continues to grow, it is becoming increasingly evident that this mechanism plays a crucial role in cancer and stem cell biology [110, 111]. However, much remains to be uncovered, particularly in the context of brain development, neurological function and diseases.

### Protein translation

In eukaryotes, mRNA translation typically depends on chemical modifications at both the 5′ and 3′ ends of linear RNAs, which enhance stability, facilitate transport, and promote protein synthesis. The 5′ end is capped with a methylated guanosine (m^7^G), connected to the mRNA via a 5′–5′ triphosphate bridge, while the 3′ end is polyadenylated, forming a poly(A) tail required for efficient protein translation [112–114]. However, some mRNAs can undergo cap-independent translation via internal ribosome entry sites (IRES), enabling protein synthesis without the need for a 5′ cap and 3′ poly(A) tail [115]. Given their covalently closed-loops structure, circRNAs lack 5′ and 3′ ends, making cap-independent translation the only feasible mechanism (Fig. 2B).

Nearly four decades ago, circRNA translation was first observed in the human hepatitis delta virus, where a circRNA containing an open reading frame (ORF) with start and stop codons directed the synthesis of a 215-amino-acid protein [116]. Subsequent studies in the mouse testis-determining gene *Sry* provided further evidence of circRNA translation [117]. Later research demonstrated that synthetic circRNA with an IRES can indeed be translated in vitro [118]. More recently, naturally occurring circRNA translation has been confirmed in vivo [119–121]. Systematic approaches combining polysome profiling, non-poly(A)-selected RNA sequencing, and bioinformatics revealed robust evidence of endogenous circRNA translation in human cells [122–124].

Several cap-independent mechanisms have been identified for circRNA translation, including IRES-dependent [122–125], short IRES-like A/U-rich sequences [126], m6A IRES (MIRES)-dependent [127, 128], and rolling circle translation (RCA)-dependent translation [119]. However, the precise mechanisms by which ribosomal subunits and translation initiation factors assemble during circRNA cap-independent translation remains unclear. Recent studies suggest that combinatorial interactions between different RBPs and components of the elF4 and elF3 complexes are crucial for the regulation of cap-independent translation [127–131].

Our current understanding of the abundance and functional roles of circRNA-derived proteins remains limited, but emerging evidence indicates that these circRNA-derived proteins play key roles in normal development and may be implicated in various diseases. For instance, in fruit flies, a circRNA from the *muscleblind* locus (circMbl) generates a peptide under starvation conditions, which localizes to synaptosome fractions in fly heads. The absence of identifiable peptide signal sequences in the proteins encoded by both the Mbl mRNA and circMbl suggests that translation may occur locally at the synapses [124]. Another fruit fly circRNA, derived from the *sulfateless* (*sfl*) gene (circSfl), produces a small protein regulated by insulin-mediated lifespan extension and aging, potentially linking circSfl-derived protein to lifespan regulation [132].

In humans, a circRNA derived from the amyloid β precursor protein (*APP*) gene, known as circAβ-a, can be translated into a novel Aβ-containing polypeptide in the brains of both Alzheimer’s disease patients and non-dementia individuals. While no evidence currently links the circAβ-a-derived protein to dementia, this discovery suggests an alternative pathway for Aβ biogenesis, which could help explain sporadic cases of the disease [126]. Other circRNA-derived proteins have primarily been associated with cancers [122–124, 127, 133]. Given that approximately 24% of mammalian circRNAs contain 3′ and/or 5′ UTRs [59], it is likely that many circRNA-derived proteins will be identified in future studies.

### Transcriptional regulation

CircRNAs can regulate gene transcription through various mechanisms. Some circRNAs interact directly with components of the RNA Polymerase II (Pol II) complex, acting as either positive or negative transcriptional regulators (Fig. 2C). For instance, a recent study demonstrated that metal-responsive element-containing circRNAs inhibit the transcription of copper stress-responsive genes by blocking the recruitment of gawky, a chromatin-interacting RBP, to active chromatin regions. This blockage leads to aberrant cytoplasmic accumulation of gawky, thus disrupting gene transcription [134].

Certain nuclear intronic circRNAs, such as ci-ankrd52, accumulate at their transcription sites, where they serve as positive regulators of their parental gene by association with Pol II complex [47]. Similarly, exon-intron circRNAs like circEIF3J and circPAIP2 have been shown to regulate Pol II transcription by forming RNA-RNA interactions with U1 small nuclear ribonucleoprotein (U1 snRNP), thereby modulating the transcription of their parental genes [38]. Another example is circHuR, which represses transcription of its parental gene, *human antigen R* (*HuR*), by directly interacting with CCHC-type zinc finger nucleic acid binding protein (CNBP). This interaction prevents CNBP from binding to the *HuR* promoter, consequently suppressing *HuR* transcription and inhibiting gastric cancer progression [135].

CircRNAs can also activate parental gene transcription through mechanisms involving intronic enhancers [136] or promoter methylation [137]. For example, in fruit flies, a maternally inherited intronic circRNA (sisR-4) activates an enhancer located within the intron of its parental *deadpan* (*dpn*) gene, which is essential for the regulation of zygotic gene expression during embryogenesis [136]. Similarly, a *FLI1* exonic circRNA (FECR1) binds to the *FLI1* promoter and recruits TET1 (Tet Methylcytosine Dioxygenase 1) to induce DNA demethylation at a CpG island in the *FLI1* promoter. This epigenetic modification enables *FLI1* to drive metastasis in breast cancer by leveraging both canonical oncogenic pathways and epigenetic regulation via FECR1 [137]. In addition to their nuclear roles in transcriptional regulation, several cytoplasmic circRNAs have been found to regulate the expression of transcription factors primarily by sponging miRNAs that target specific transcription factors [138–140].

### RNA binding protein transport, scaffold, and decoy

Several studies suggest that a subset of circRNAs may play critical roles in transporting RBPs to specific subcellular locations, facilitating the assembly of protein-RNA and enzyme-substrate complexes, as well as acting as protein decoys or sponges (Fig. 2D). These mechanisms are closely linked to various aspects of circRNA biogenesis, localization, stability and degradation [50, 51, 141, 142]. CircRNAs can act as mediators in biochemical pathways, selectively transporting, scaffolding, or sponging molecules, thus forming complex regulatory networks (Fig. 2E). These overlapping mechanisms converge to produce context-specific functional outputs in diverse cellular processes.

For example, in colorectal cancer, the upregulation of circYAP1 is linked to a reduction in immune activation against cancer cells. CircYAP1 binds directly to the YAP1 (yes1 associated transcriptional regulator) protein, preventing its phosphorylation which enhances YAP1 nuclear import, where interactions with transcription factor 4 (TCF4) promotes the expression of the immune checkpoint inhibitor PD-L1 (CD274), leading to immune evasion and tumor progression [143]. Similarly, circAMOTL1, which is highly expressed in neonatal human cardiac tissue, promotes AKT-mediated cardiomyocyte survival and repair. Research indicates that circAMOTL1 binds to both PDK1 (pyruvate dehydrogenase kinase 1) and AKT1 (AKT serine/threonine kinase 1), enhancing AKT1 phosphorylation and facilitating the nuclear translocation of pAKT1, thereby promoting cardioprotective effects [144]. In glioblastoma, reduced miRNA abundance compared to normal brain tissue has been associated with an aberrant nuclear localization of DICER1 (dicer 1 ribonuclease III, a crucial endonuclease for miRNA maturation). This mislocalization could be mediated by its interaction with RNA Binding Motif Protein 3 (RBM3) and circ2082, one of the most upregulated circRNAs in glioblastoma cells [145]

CircRNAs also act as scaffolds or recruiters to modulate protein degradation. In hepatocellular carcinoma, circPABPC1 directly links integrin subunit beta 1 (ITGB1) to the 26S proteasome for degradation in a ubiquitination-independent manner [146]. In breast carcinoma, circDNMT1 expression is increased and binds to the RBP AUF1 (HNRNPD, heterogeneous nuclear ribonucleoprotein D) and the transcription factor TP53 (tumor protein P53), promoting nuclear translocation of both proteins, which enhances cell proliferation and inhibits of senescence [147]. In non-cancer mouse cells, circFoxo3 binds to the cell cycle-associated proteins CDK2 (cyclin dependent kinase 2) and p21 (CDKN1A, cyclin dependent kinase inhibitor 1A), reducing the formation of cyclin E/CDK2 complexes, thereby blocking the G1 to S phase transition in the cell cycle [148].

### Competition between linear and circular RNA expression

CircRNAs are co-transcriptionally produced with their cognate mRNAs and can act as transcript regulators affecting the expression of their linear counterparts (Fig. 2F). Investigations into this competitive dynamic have revealed that the balance between circular and linear RNA production can significantly impact gene expression and function. One notable example involves circMbl and its cognate mRNA. The biogenesis of circMbl is tightly regulated by a splicing factor encoded by the *muscleblind* (*Mbl*) gene that binds to specific sites in the flanking introns of circMbl. This interaction establishes a feedback loop, as circMbl directly binds to MBL protein, modulating its availability and consequently controlling its own production. This mechanism exemplifies how circRNA can act as a regulator of its associated splicing factor, maintaining cellular homeostasis [9].

Recent findings have introduced a novel mechanism by which circRNAs can regulate mRNA stability. CircRNAs that bind both the exon junction complex (EJC) and the 3′ UTR of mRNAs have been shown to induce degradation of the bound mRNA. This process resembles the canonical nonsense-mediated decay (NMD) and relies on EJC binding downstream of termination codons and NMD factors such as PNRC2 (Proline Rich Nuclear Receptor Coactivator 2), UPF1 (UPF1 RNA Helicase and ATPas) and UPF2 (UPF2 Regulator of Nonsense Mediated mRNA Decay). In the circRNA-mediated pathway, circRNAs tethered to the 3′ UTR bring EJCs into proximity, facilitating mRNA degradation through an NMD-like mechanism. The efficiency of this process depends on the number and location of circRNA-mRNA binding sites, illustrating the complexity of circRNA-mediated regulation [149].

Another interesting case is circHOMER1, which has strong sequence complementarity to the 3′ UTR of HOMER1B mRNA isoform. Reduced expression of circHOMER1 has been linked to increased HOMER1B expression, suggesting a regulatory interplay between circular and cognate linear RNA. The RBP ELAVL4 binds to sites near the complementary regions on both the circHOMER1 and the HOMER1B 3′ UTR, suggesting the role of circHOMER1 in regulating linear HOMER1B expression [150].

## Computational identification of circRNAs

Recent advances in understanding circRNA biogenesis and their roles in various pathological and physiological contexts have driven the development of bioinformatic tools for circRNAs analysis [43, 151]. Over three million unique circRNA transcripts across multiple species have been catalogued, with online databases such as circBase [152], circBank [153], CIRCpediav2 [154], and circAtlas3 [155], available for reporting and comparison. These resources provide comprehensive information including associated studies, cell types, chromosome coordinates, predicted splice sites, RNA-binding proteins sites, and miRNA binding sites. Specialized resources like NeuroCirc, which focuses on circRNAs in the human brain [156], are particularly useful for researchers studying neuronal tissues.

The main strategy for identifying circRNAs from short-read RNA sequencing data relies on detecting backsplice junctions (BSJs). Given that circRNAs lack a poly(A) tail, cDNA library preparation methods must avoid poly(A) selection. Instead, these methods typically involve ribosomal RNA depletion followed by cDNA synthesis that captures both coding and non-coding RNAs, including circRNA molecules. To date, all computational tools for circRNA identification rely on initial experimental evidence from RNA-seq data, with fully de novo computational methods based solely on genomic features yet to be developed. Current computational approaches to circRNA identification are divided into two main strategies: pseudo-reference-based and chimeric-read-based [157, 158].

Pseudo-reference-based methods use existing gene annotation data to create BSJ references, which can limit discovery in less-studied cell types or species. Notable pseudo-reference-based tools include Circall [159], NCLscan [160], and KNIFE [161]. For example, Circall [159] implements a two-step pseudo-reference approach: it first maps RNA-seq reads to an annotated reference transcriptome to remove linear RNA reads, then aligns the remaining unmapped reads to a BSJ reference database. This database is constructed from annotated RNA sequences, creating pseudo-sequences for circRNAs and their tandem counterparts by combining sequences from constituent exons. To reduce false positives, Circall applies a two-dimensional local false discovery rate (2dFDR) method [162], which accounts for both BSJ-supporting reads and circRNA length. Chimeric-read-based methods align sequencing reads directly to a reference genome, using noncolinear or chimeric reads to detect BSJs. For instance, circRNA_finder [11] utilizes the chimeric alignment output from STAR program [163] to identify chimeric junctions on the same chromosome flanked by canonical donor-acceptor splice sites (GT—AG). Other chimeric alignment tools include CIRI2 [164], DCC [165] and CIRCexplorer3 [166]. In the case of CIRI2 [164], it reports BSJ mapped reads from alignments using BWA-MEM program [167].

There is currently no single “gold standard” toolkit for circRNA identification [168, 169], and combining multiple detection methods often enhances accuracy. A notable evaluation of 16 circRNA detection tools identified over 3,15,000 unique circRNAs, with 1516 validated experimentally by RT-qPCR, demonstrating the value of a consensus approach [169]. Examples of multi-tool platforms include SRCP [170], CirComPara2 [171] and nf-core/circRNA [172]. Long-read sequencing platforms, such as Oxford Nanopore Technology, have recently advanced circRNA identification [173] by enabling full circRNA transcript coverage and detection of isoforms [174] as well as native RNA modifications [175]. Unlike short-read sequencing, which limits full transcript identification and quantification, long-read sequencing technologies provide a clearer picture of co-regulated transcripts and insights into splicing processes [176].

To investigate miRNA sponging potential, traditional miRNA target prediction tools like miRanda [177], TargetScan [178], and RNAhybrid [179] have been repurposed for circRNA-miRNA interaction prediction [41, 155, 180]. For circRNA-RBP interaction prediction, tools like catRAPID omics v2.0 integrate experimentally validated RNA-binding proteins using motifs collected from multiple databases [181]. Additional resources for circRNA-RBP interaction prediction include CircSSNN [182], CRAFT [183], CircInteractome [41], CircSite [184], and circAtlas [155]. For predicting the protein-coding potential of circRNAs, CircProPlus employs unsupervised learning algorithm and logistic regression model based on open reading frame (ORF) characteristics such as ORF size and coverage [185]. Other useful tools include cirCodAn [186], C2CDB [187], CRAFT [183] and circRNADb [188]. Visualization tools such as CIRI-vis [189] and circView [190] further support the analysis of splicing patterns, regulatory elements, and miRNA/RBP binding sites, advancing our understanding of circRNA functionality and complexity.

## Experimental validation and characterization of circRNAs

Identifying circRNAs in RNA-seq data is a valuable initial step in selecting candidates for further experimental validation and characterization. Quantitative reverse transcriptase polymerase chain reaction (qRT-PCR) remains one of the most widely used methods for analyzing circRNA candidates [169, 191–193]. This approach uses divergent primers specifically designed to amplify BSJs with primer design principles akin to mRNAs, however it is also essential to know the candidate BSJ sequence and its flanking regions. Tools such as circPrimer2 [194] and CircInteractome [41] assist with primer design for known BSJs. For novel circRNA candidates not catalogued in public databases, general-purpose tools like Primer3 [195] can be used by providing a sequence spanning the BSJ to generate divergent primers and predict amplicon sizes. Sanger sequencing also serves to confirm backsplice junctions via PCR with divergent primers targeting candidate circRNAs [196, 197].

Northern blotting is a commonly used method for circRNA validation. Probes are designed to target BSJs via hybridization with complementary RNA sequences that are separated by size in an electrophoresis assay [29, 45, 46, 85, 100, 101]. Additionally, treatment with the 3–5′ exonuclease RNase R can enrich circRNA levels by selectively digesting linear RNAs, which is beneficial prior to RNA-seq, qRT-PCR, and northern blot experiments, as circRNAs are often expressed at low levels [10, 193, 198]. Nonetheless, highly structured regions in linear RNA may block RNase R digestion, potentially confounding this interpretation [198].

Given that circRNAs are often significantly less abundant than their linear cognate RNAs, determining their subcellular localization is crucial for understanding their potential function [94, 199]. The quantification of circRNAs by qRT-PCR in different cellular fractions such as the nucleus [47], ribosomes [199], mitochondria [17], and exosomes [69, 70] can reveal clues about their roles, providing hypotheses for further characterization. However, subcellular fractionation techniques may suffer from purity issues and potential contamination. RNA fluorescence in situ hybridization (FISH) using antisense probes targeting the BSJs offers a more precise method for single-cell localization. Despite the high accuracy, detecting circRNAs by FISH can be challenging due to their low abundance, complex secondary structures, or protein interactions at the BSJ. Once circRNAs are validated and their subcellular localization determined, appropriate experimental approaches can be selected for further characterization. For instance, nuclear circRNAs are most likely almost all non-protein-coding and may serve regulatory roles, cytoplasmic circRNAs have been shown to function as miRNA sponges, RBP transporters, or scaffolds while ribosome-associated circRNAs may indicate potential translation into peptides.

To further investigate the functional role of circRNAs, modulation of their expression through knockdown, knockout or overexpression assays provides valuable functional information. Specifically, for loss-of-function studies, circRNAs can be knocked down via small hairpin RNA (shRNA), small interfering RNA (siRNA) [200, 201], or CRISPR-Cas13 [199, 202] targeting the BSJs. Yet, effective circRNA depletion without disrupting their linear RNA counterparts remains a major challenge, as circRNAs are predominantly derived from protein-coding genes. In cases where circRNAs lack a cognate linear RNA [89], are formed by intronic complementary sequences [85, 203, 204] or specific RBP binding sites, knockout approaches may be more feasible. CircRNA overexpression can also elucidate their functional roles, via strategies such as expression vector construction with circRNA-producing exons and their flanking introns [46, 70, 100, 205, 206] or transfecting cells with synthetic circRNAs generated through in vitro transcription and circularization [207–209]. A thorough discussion of all available validation and characterization methods and their limitations is beyond the scope of this review; therefore, we recommend several comprehensive studies for greater comprehension [8, 210, 211].

## CircRNAs in the brain and neurological conditions

The current literature extensively demonstrates new mechanisms and functional roles of circRNAs in cancer biology. However, evidence regarding the mechanism of circRNAs in specific brain cells and human neurological disorders remains limited. Nevertheless, compelling findings highlight the critical importance of RNA splicing in brain development and disease [212, 213], with growing research suggesting circRNAs as an essential regulatory layer in neuronal tissue and associated disorders [26, 28, 29, 83, 84, 214]. Studies have consistently shown that circRNA expression levels in the brain are significantly higher than in other tissues, a pattern observed across various species, including humans [29], mice [84], rats [215], and fruit flies [11].

CircRNAs are not only highly abundant in the brain [28, 29, 156, 216] but also present a dynamic range of expression across cell types, development stages, and aging [11, 26, 215, 217]. In neurons, genes encoding synaptic proteins tend to produce more circRNAs, many of which are enriched in the synaptosome, suggesting potential roles in neuronal differentiation, synaptic function, and plasticity [26, 83, 84, 218]. CircRNAs accumulate in the central nervous system with age [11, 32, 215, 217, 219], potentially contributing to cell senescence and age-related neurological disorders [32, 217, 219]. Dysregulation of circRNA expression and function has been implicated in several neurological disorders, with much of this research emerging over the past decade [26, 214, 220–223]. Despite these advances, the precise roles of circRNAs in the pathology of neuropsychiatric and neurodegeneration disorders remain poorly understood.

This review focuses on three neuropsychiatric disorders (schizophrenia, bipolar disorder, and major depressive disorder) and two neurodegenerative diseases (Alzheimer’s disease and Parkinson’s disease), summarizing what is currently known about circRNAs in these conditions. Table 1 presents a concise overview of key findings with some experimental evidence provided in relevant published studies on human circRNAs, which are further explored in the following sections.

**Table 1:** Summary of select studies describing dysregulated circRNAs in neurological disorders including schizophrenia (SCZ), bipolar disorder (BD), major depressive disorder (MDD), Alzheimer’s disease (AD), and Parkinson disease (PD).

Condition	circBase ID	CircRNA parent gene symbols	Source	Sample size	Methods	Overall dysregulation of circRNAs and/or key finding	Ref
SCZ	hsa_circ_0000638 hsa_circ_0005035 hsa_circ_0001200 hsa_circ_0001789 hsa_circ_0007762 hsa_circ_0005035	*ETFA* *IGFR1* *PTTGIP* *RAB1FIP1* *STXBP5* *IGF1R*	Plasma	8 CON 10 SCZ	RNAseq	1 upregulated 234 downregulated	[232]
SCZ	hsa_circ_0003999 hsa_circ_0030042	*MPND* *FOXO1*	Blood	3 CON 3 SCZ	RNAseq RT-qPCR	392 upregulated 58 downregulated	[233]
SCZ/BD	hsa_circ_0005813 hsa_circ_0001307 hsa_circ_0080653 hsa_circ_0099001 hsa_circ_0003290 -- hsa_circ_0000914 hsa_circ_0001550	*LONP2* *RAD54L2* *NCF1C* *SCARNA10* *TMEM2* *EZH1* *FKBP8* *RARS*	Blood	20 CON 19 BP 20 BD	RNAseq	7 upregulated in BD vs CON 26 downregulated in BD vs CON 8 upregulated in SCZ vs CON 14 downregulated in SCZ vs CON	[234]
SCZ	hsa_circ_0039066	*PRR14*	Blood	50 CON 50 SCZ	RNAseq	13 downregulated	[235]
SCZ	hsa_circ_0141293 hsa_circ_0002672 hsa_circ_0112551 hsa_circ_0002602 hsa_circ_0073237 hsa_circ_0115215 hsa_circ_0002506 hsa_circ_0000023 hsa_circ_0002799 hsa_circ_0084780 hsa_circ_0141280 hsa_circ_0142301 hsa_circ_0104963 hsa_circ_0007178	*LONP2* *RABGAP1* *GPR137B* *PPP2CA* *VCAN* *TOP1* *MYO9A* *HP1BP3* *ZNF236* *STAU2* *SV2B* *STXBP5* *IGF1R* *BRAF*	DLPFC	20 CON 20 SCZ	RNAseq RT-qPCR	184 upregulated 390 downregulated	[236]
SCZ	hsa_circ_0120251 hsa_circ_0137107 -- hsa_circ_0130962 hsa_circ_0005035 hsa_circ_0006961 hsa_circ_0007661	*NRXN1* *STAU2* *SV2B* *STXBP5* *IGF1R* *BRAF* *RABGAP1*	DLPFC	178 CON 171 SCZ	RNAseq	182 downregulated 10 upregulated	[237]
BD/SCZ	hsa_circ_0006916 hsa_circ_0030788	*HOMER1* *NALCN*	OFC	34 CON 34 SCZ 32 BD	Microarray RT-qPCR	38 dysregulated in BD vs CON 34 dysregulated in SCZ vs CON	[223]
BD	-- hsa_circ_0141109 -- -- hsa_circ_0128413 hsa_circ_0100444	*Chr7:141760111-141786128* *DLEU2* *Chr6:29945234- 30009177* *chr19:54726815-54784154* *SGCD* *LOC100616668*	Blood	20 CON 20 BD	RNAseq RT-qPCR	44 downregulated 50 upregulated	[253]
BD	hsa_circ_0056537	*CCNT2*	ACC; B-LCL	27 CON 24 BD 12 CON 19 BD	RNAseq RT-qPCR	26 dysregulated, and circCCNT2 is associated with bipolar disorder and lithium treatment	[254]
MDD	--	*DYM*	Plasma	32 CON 49 MDD	RT-qPCR	CircDYM could be a potential biomarker for MDD diagnosis as well as determination of transcranial magnetic stimulation effectiveness in treating MDD	[221]
MDD	hsa_circ_0015067	*PBX1*	Venous blood	6 CON 6 MDD	RNAseq	Intersection of homologous circRNAs between human GSE182193 dataset and CUMS mouse model: 14 upregulated 1 downregulated	[262]
MDD	--	*DYM*	Serum	30 CON 50 MDD	RT-qPCR	CircDYM could be a potential therapeutic for treating MDD through microglial functional modulation through circDYM-mir9-HECTD1/HSP90 axis.	[266]
AD	-- -- -- --	*HOMER1* *KCNN2* *ICA1* *FMN1*	Parietal cortex; IFG; APC; STG; Perirhinal cortex	13 CON 83 AD 195 samples (40 controls,89 definite AD,31 probable AD, and 35 possible AD)	RNAseq RT-qPCR	236 significant ADAD-associated circRNAs	[278]
AD	hsa_circ_0003391	*UBASH3B*	Plasma; Blood	50 CON 50 AD 20 Dementia with Lewy Bodies 40 Vascular Dementia	Microarray RT-qPCR	High positive correlation between multiple AD clinical traits and circUBASH3B expression	[282]
AD	hsa_circ_0020462 hsa_circ_0139156 -- hsa_circ_0135879 hsa_circ_0127616 hsa_circ_0110235 hsa_circ_0006852	*DOCK1* *NTRK2* *DLG1* *TRAPPC9* *APC* *KIF1B* *CORO1C*	Frontal lobe; Hippocampus; Temporal Cortex; Plasma	222 CON 213 AD	RNAseq	Using six different publicly available datasets of RNA sequencing data, a select grouping of circRNAs were dysregulated among AD individuals across different brain regions along with plasma samples, some which had host genes implicated in AD pathophysiology	[283]
AD	hsa_circ_0001481 hsa_circ_0001819	*EMB* *UBR5*	Blood	17 CON 11 aMCI 22 SCD 5 AD	Microarray RT-qPCR Luciferase assay	Potential biomarkers identified for early detection of AD from blood	[284]
AD	hsa_circ_0014353 hsa_circ_0014356 hsa_circ_0074533 hsa_circ_0077001 hsa_circ_0022417 hsa_circ_0089894	*RPS27* *RPS27* *RPS14* *EEF1A1* *FTH1* *TMSB4X*	Blood	139 CON 143 AD 50 VaD 51 PDD 52 bvFTD 50 DLB	RNAseq	A panel of six circRNAs were developed from a combination of three datasets to measure blood derived circRNAs for diagnosis of AD and correctly differentiate from other dementia types.	[285]
AD	hsa_circ_0050263 -- hsa_circ_000302 -- --	*ATP13A1* *BRAF* *PTRM1* *TADA2A* *NOL10*	Blood	29 CON 26 AD 5 MCI	Microarray RT-qPCR	Five significantly dysregulated circRNAs were validated and had good diagnostic accuracy for distinguishing AD.	[286]
AD	-- -- --	*HOMER1* *GSK3β* *GSK3β*	Frontal lobe; Temporal lobe	20 CON 19 AD	RNAseq Proteomics Synaptosome confocal microscopy	circRNA mislocalization in AD pathophysiology demonstrated by enrichment of circRNAs at synapses	[287]
AD	hsa_circ_0006916 hsa_circ_0073128 hsa_circ_0000437 hsa_circ_0127664 hsa_circ_0006837 hsa_circ_0127521	*HOMER1* *HOMER1* *CORO1C* *KCNN2* *RERE* *MAN2A1*	APC; STL; PHG; IFG		RNAseq	Across all clinical dementia rating stages at FDR of greater than or equal to 0.05 in all four brain regions, 147 dysregulated circRNAs were identified, with only 4 dysregulated in all four brain regions	[288]
AD	hsa_circ_0073127 hsa_circ_0005494 hsa_circ_0001911 hsa_circ_0117996	*HOMER1* *ATP13A3* *FANCB* *FASTKD1*	DLPFC	8 CON 9 AD	RNAseq	120 upregulated 1325 downregulated	[289]
AD	hsa_circ_0008521	*PSEN1*	Entorhinal cortex; Hippocampus Postcentral gyrus; SFG	54 AD 62 AD 68 AD 69 AD	Microarray	CircRNAs derived from PSEN1 were shown to have predictive influences on molecular mechanisms of AD progression	[290]
AD	hsa_circ_0008521 hsa_circ_0003848	*PSEN1* *PSEN1*	Parietal cortex; Frontal pole; STG; PHG; IFG	23 CON 21 ADAD 253 AD	RNAseq	Higher circPSEN1 expression is specific to ADAD and independent of mutation	[294]
PD	hsa_circ_0001776 hsa_circ_0000944 hsa_circ_0002627 hsa_circ_0142640 hsa_circ_0004368 hsa_circ_0001187 hsa_circ_0004833 hsa_circ_0000497 hsa_circ_0000069 hsa_circ_0000994	*ESYT2* *CCDC9* *BMS1P1* *INTS6L* *REPS1* *DOP1B* *AR6IP1* *SLAIN1* *STIL* *SLC8A1*	Blood	PPMI 161 CON 259 PD ICICLE- 48 CON 48 PD	RNAseq	Three significant circRNAs in PPMI and not statistically significant differentially expressed circRNAs after correction in ICICLE-PD but replication of the direction of 7 previously found significant circRNAs were identified.	[305]
PD	hsa_circ_0065842 hsa_circ_0067965 hsa_circ_0131191 hsa_circ_0085965 hsa_circ_0138219 hsa_circ_0093281 hsa_circ_0034762 hsa_circ_0037165 hsa_circ_0041511 hsa_circ_0115568	*GNAI2* *FNDC3B* *chr6:158675463-158675686* *SHARPIN* *VAV2* *chr10:20571145-20571330* *MAPKBP1* *MRPL28* *ZZEF1* *chr20:62034919-62035057*	Plasma	3 CON 9 PD	RNAseq	1005 downregulated	[306]
PD	-- -- -- -- -- -- -- -- -- -- -- -- --	*AFF2* *ITGAX* *SPI1* *NCF1* *PADI4* *ETFA* *ESYT2* *FAM13B* *RNF13* *CCDC91* *SUZ12* *CSEL1* *SHOC2*	Blood	PDBP 460 CON 717 PD PPMI 143 CON 528 PD	Bioinformatics	71 circRNAs could distinguish between genetic PD and at-risk participants with an AUC of 0.954 and 0.929 respectively.	[307]
PD	hsa_circ_0000061 hsa_circ_0003258	*SCHM1* *ZNF652*	Exosomes	15 CON 23 PD	RNAseq RT-qPCR	circSCMH1 expression had strong correlation between MDS-UPDRS III scores in PD patients and could potentially regulate ARID1A and C1orf115 function.	[308]
PD	-- hsa_circ_0036353 hsa_circ_0000690 hsa_circ_0001535 hsa_circ_0001451 hsa_circ_0004870 hsa_circ_0000605 hsa_circ_0014606 hsa_circ_0001801 hsa_circ_0001772	*HBB* *SIN3A* *ITGAL* *FAM13B* *FBXW7* *RBM39* *SLTM* *YY1AP1* *PCMTD1* *RBM33*	Blood	4 CON 4 PD	RNAseq	282 downregulated 129 upregulated	[309]
PD	hsa_circ_0000497 hsa_circ_0001187 hsa_circ_0004368 hsa_circ_0001566 hsa_circ_0003848 hsa_circ_0006916	*SLAIN1* *DOP1B* *REPS1* *MAPK9* *PSEN1* *HOMER1*	Blood	60 CON 60 PD	RT-qPCR	6 downregulated	[310]
PD	hsa_circ_0127305	*SNCA*	MPP^+^-induced PD cell models	--	RT-qPCR Dual luciferase assay MTT assay	CircSNCA indirectly regulates miR-7 and SNCA	[311]
PD	hsa_circ_0000994	*SLC8A1*	Amygdala; MTG; Substantia nigra	27 CON 42 PD	RNAseq RT-qPCR	Significant difference in substantia nigra of PD and control, with circSLC8A1 upregulated in this region in PD and having potential sponging of miR-128. CircRNA expression in brain regions studied found to be inversely proportional to RNA editing levels	[313]
PD	hsa_circ_0060180	*DLGAP4*	MPP^+^-induced PD cell models	--	Cell assays Luciferase assay RT-qPCR	CircDLGAP4 attenuated effects if MPP+ neurotoxin in SH-SY5Y and MN9D treated cells.	[314]
PD	hsa_circ_0004846	*SAMD4A*	MPP^+^-induced PD cell models	--	Cell assays Luciferase assay RT-qPCR	CircSAMD4A regulates miR-29c-3p and knockdown of this circRNA induces protective effect on MPP treated SH-SYBY cells	[315]
PD	--	*BACH1* *GDI2* *TMEM138* *EPS15* *JA423830* *LINC012333*	Blood	40 CON 40 PD	RNAseq	circEPS15 is a predicted target for PD treatment	[317]
PD	hsa_circ_0000284	*HIPK3*	Serum; CSF	95 CON 92 PD	RT-qPCR RNA IP assay Luciferase assay	circHIPK3 sponges miR-124 which disrupts STAT3/NALP3 pathway in PD leading to neuroinflammation	[319]
PD	hsa_circ_0054220	*LOC728730*	MPP^+^-induced PD cell model	--	Cell assays Luciferase assay RT-qPCR	Inhibition of circLOC728730 suppressed apoptosis in MPP treated cells and was found to bind to two miRNAs (miR-145 and miR-625) which regulates HMGA1	[320]
PD	hsa_circ_0007021	*HIVEP2*	MPP^+^-induced PD cell model; Venous blood	85 CON 127 PD	Cell assays Luciferase assay RT-qPCR	Increasing circHIVEP2 attenuates MPP induced inflammation and apoptosis. CircHIVEP2 was also found to bind miR-485-3p which when increased had similar effect to increasing circHIVEP2 but exact mechanism of action unknown.	[321]
PD	hsa_circ_0004381 hsa_circ_0017204 hsa_circ_0085869 hsa_circ_0090668	*ARID1B* *TCONS_l2_00002816* *FAM83H* *HUWE1*	Plasma	100 CON 300 PD	Microarray RT-qPCR	A small panel of circRNAs could predict early onset PD and distinguish late PD from early	[322]

circBase IDs [152] are provided when reported in the original study. RNA source encompasses cell-types or tissue used for identification of circRNAs. Differentially expressed circRNAs are indicated by their host gene symbol: downregulated circRNAs are shown in underlined red, while upregulated circRNAs are shown in slanted blue.

*CON* controls, *DLPFC* dorsolateral prefrontal cortex, *OFC* orbital frontal cortex, *ACC* anterior cingulate cortex, *IFG* inferior frontal gyrus, *APFC* anterior prefrontal cortex, *STG* superior temporal gyrus, *PHC* parahippocampal cortex, *MTG* medial temporal gyrus, *STL* superior temporal lobe, *CSF* cerebrospinal fluid, *B-LCL* B lymphoblastoid cell line.

### Schizophrenia

Schizophrenia is a complex polygenic neurological disorders characterized by a spectrum of symptoms [224, 225]. The disorder typically manifests between the ages of 16 and 30, with an earlier onset and higher prevalence in males compared to females [224, 226, 227]. Symptoms include hallucinations, delusions, impaired emotional expression, and disorganized speech [224, 227, 228]. A recent genome-wide association study (GWAS) identified 287 genomic loci associated with schizophrenia with at least 600 genes potentially implicated in schizophrenia [229], in which the majority of genetic variants occurred in non-coding regulatory regions of the genome [229, 230], highlighting the importance of the non-coding genome in understanding complex disorders like schizophrenia. Investigating the role of circRNAs in schizophrenia offers a promising perspective on how non-coding RNAs may contribute to disease pathophysiology [26, 231]. Although the research field is still in its early stages, studies have reported altered circRNA expression in both peripheral blood samples [232–235] and postmortem brain tissues [223, 236, 237] from schizophrenia patients.

CircRNAs in blood cells are particularly intriguing as potential biomarkers or therapeutic targets; For example, one study found 22 differentially expressed circRNAs, with 14 downregulated and 8 upregulated in disease [234]. Another study reported 13 downregulated circRNAs compared to healthy controls [235]. In early-onset schizophrenia patients, circRNA expression was dramatically reduced, with 234 downregulated circRNAs and one upregulated [232]. Conversely, a smaller cohort study reported 392 upregulated and 58 downregulated circRNAs in schizophrenia patients compared to controls [233]. There is no overlap for genes reported in these studies which underscores the challenges of biomarker discovery in schizophrenia.

**Fig. 3:**
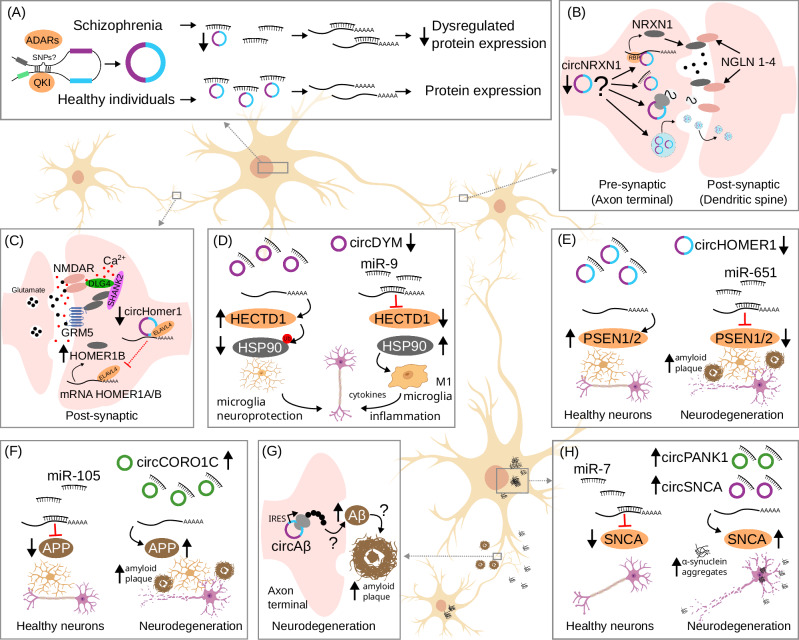
Dysregulated circRNAs may disrupt different mechanisms associated with disease. (**A**) Dysregulated circRNA biogenesis in schizophrenia may arise from altered regulation of RBPs due to schizophrenia-associated genetic variants in intronic regions, affecting circRNA expression and downstream miRNA-mediated gene regulation. (**B**) Reduced CircNRXN1 expression may disrupt synapse formation and transmission through several potential mechanisms, including impairments in RBPs or mRNA transport/scaffolding, altered miRNA sponging, peptide translation, or modifications in intercellular communication by exosomes. However, these hypotheses remain to be tested. (**C**) Reduced circHOMER1 expression in bipolar disorder is linked to loss of cognitive flexibility through a complex network of interactions affecting glutamatergic synaptic transmission. A similar mechanism may also play a role in schizophrenia and other neurological disorders. (**D**) In major depressive disorder, reduced circDYM expression increases miR-9 bioavailability, leading to downregulation of HECTD1. This, in turn, inhibits HSP90 ubiquitination, resulting in microglial activation and enhanced inflammation by pro-inflammatory cytokines. (**E**) Reduced circHOMER1 expression in Alzheimer’s disease patients is proposed to increase miR-651 bioavailability, leading to inhibition of PSEN1 and PSEN2 expression. This dysregulation may contribute to amyloid plaque accumulation and neurodegeneration. (**F**) Increased circCORO1C expression in Alzheimer’s disease patients reduces miR-105 bioavailability, resulting in upregulation of *APP* expression and contributing to amyloid plaque formation and neurodegeneration. (**G**) CircAβ, derived from the *APP* gene, can be translated into peptide sequences that may contribute to accumulation of amyloid plaque accumulation and neurodegeneration in Alzheimer’s diseases. However, the mechanism by which these circRNA-derived peptides are exported and their contribution to amyloid plaque formation and dementia remains unclear. IRES internal ribosome entry site. (**H**) Increased expression of circPANK1 and circSNCA in the substantia nigra of Parkinson’s disease patients is associated with their function as miR-7 sponges. This increases *SNCA* (α-synuclein) expression, promoting the formation of neurotoxic aggregates in dopaminergic neurons.

In postmortem brain tissue, particularly the dorsolateral prefrontal cortex (DLPFC), circRNA expression is significantly reduced in schizophrenia patients compared to controls. A recent study identified 574 differentially expressed circRNAs, with 184 upregulated and 390 downregulated [236]. The authors proposed that schizophrenia involves dysregulated circRNA biogenesis, characterized by a global reduction in circRNA levels in patients’ brains. Another study found 203 differentially expressed circRNAs in the DLPFC of patients, of which 182 (90%) were downregulated [237]. These findings support the hypothesis proposed by Mahmoudi et al. [236]. This reduction may increase the bioavailability of miRNAs, amplifying their inhibitory effects on target mRNAs and dysregulating protein expression (Fig. 3A). Two key RBPs involved in circRNA biogenesis, ADAR1 and QKI, exhibit distinct expression patterns in schizophrenia patients [238, 239], suggesting their potential involvement in impaired circRNA production. Additionally, schizophrenia-associated genetic variants in intronic regions may disrupt canonical splicing sites or complementary sequences required to form secondary structures in intronic stem-loops, which are critical for circRNA circularization [240]. Despite these findings, the precise mechanisms underlying this disruption in circRNA biogenesis and its downstream functional consequences contributing to disease remains largely unknown.

In reviewing the published data [236, 237], it was revealed that circRNAs derived from 32 genes were consistently reduced in DLPFC of schizophrenia patients. These circRNAs are derived from genes implicated in key neuronal processes, such as synapse assembly and transmission (*NRXN1, STAU2, SV2B*), synaptic vesicle exocytosis (*SV2B, STXBP5*), axon regeneration (*BRAF, IGF1R*), and insulin-like receptor signaling pathway (*RABGAP1, IGF1R*). The reduced levels of circRNAs decrease their miRNA sponging efficiency, in turn increasing bioavailable miRNAs that more effectively bind to target mRNAs, thereby repressing protein expression of genes involved in multiple biological pathways. Beyond serving as miRNA decoys, dysregulated circRNAs in schizophrenia may disrupt additional cellular processes, such as subcellular localization and transport of RBPs, potentially impairing neuronal differentiation and synaptic function. Furthermore, some circRNAs may encode peptides with unknown functions, adding further complexity to their contribution to schizophrenia risk. These disruptions, resulting from altered circRNA expression, likely contribute to broader functional impairments in neuronal processes that are central to the pathophysiology of schizophrenia.

The *neurexin 1* (*NRXN1)* gene encodes a presynaptic cell adhesion protein that interacts with a diverse repertoire of postsynaptic proteins, playing essential role in the assembly and maturation of synapses [241]. *NRXN1* encodes two main isoforms with several alternative splicing specifying different properties of synapses [242]. Several studies have identified recurrent structural genetic variations within the *NRXN1* locus in schizophrenia patients, including intronic deletions and other mutations [243–245]. These genetic variations may disrupt sequence patterns recognized by RBPs like QKI or ADARs, potentially changing the kinetics of circRNA and mRNA production. Thus, the dysfunctions associated with *NRXN1* mutations in schizophrenia may also extend to the regulatory roles of NRXN1 circRNAs, further contributing to dysregulation of synapse formation and transmission (Fig. 3B). Interestingly, the *insulin-like growth factor 1 receptor* (*IGF1R*) gene encodes a tyrosine kinase activated by a hormone called IGF1 similar in chemical structure to insulin. IGF1R circRNAs were also found to be reduced in the peripheral blood of schizophrenia patients [232], suggesting its potential as blood-based marker with similar expression profile in brain tissue. IGF1R mRNA was found to be reduced in brain tissue from subventricular zone of schizophrenia patients potentially impairing the ability of neural stem and neuronal progenitor cells to respond to IGF1 during neurogenesis [246].

A recent study identified reduced expression of a circRNA derived from *HOMER1* gene (circHOMER1) in the DLPFC of patients with schizophrenia and bipolar disorder [223]. In this study, circRNAs were detected and quantified using a circRNA microarray panel containing 13,617 probes targeting backsplice junctions, designed based on multiple RNA-seq datasets CircHOMER1 was prioritized for functional validation in mouse models and human neuronal cultures derived from patient-induced pluripotent stem cells (iPSCs). In these iPSC-derived neuronal cultures, circHOMER1 expression was consistently reduced. Functional studies in mouse models revealed that circHOMER1 competes with the HOMER1B mRNA isoform, which encodes a protein essential for synaptic plasticity and glutamate neurotransmission. The HOMER1B protein interacts with Group1 metabotropic glutamate receptors (GRM5) and anchoring proteins such as SHANK2 (SH3 and multiple ankyrin repeat domains 2) to regulate calcium signaling in excitatory synapses via N-Methyl-D-aspartate receptors (NMDAR) [247, 248]. Additionally, circHOMER1 was shown to directly interact with the 3′UTRs of HOMER1B isoform, as well as with ELAVL4, an RBP predominantly expressed in differentiated neurons. ELAVL4 is crucial for the transport and synaptic localization of circHOMER1 [150, 223] (Fig. 3C). Notably, circHOMER1 expression was consistently reduced in the DLPFC and orbitofrontal cortex (OFC) of bipolar disorder patients, suggesting potential shared mechanisms between schizophrenia and bipolar disorder. Its functional roles are further discussed in the following section, focusing on insights derived from mouse and human stem-cell-based assays.

### Bipolar disorder

Bipolar disorder (BD) is a highly heritable and complex polygenic condition characterized by diverse symptoms, including mania, depression, and hypomania. The intricate nature of bipolar disorder represents a significant challenge for the development of appropriate molecular and genetic models [249–251]. A recent GWAS involving 41,917 BD cases and 371,549 controls identified 64 loci associated with the disorder [252]. As with other GWAS findings, most identified SNPs are in non-coding regions (94%), with a substantial proportion in intronic regions (64%), which may influence the regulation of circRNA expression [252]. These regulatory SNPs could potentially lead to the gain or loss of circRNA function by affecting their biogenesis or interactions with mRNAs.

A recent study reported 33 differentially expressed circRNAs (26 downregulated and 7 upregulated) in the peripheral blood cells of 19 BD patients compared to 20 unaffected controls [234]. Another study identified 94 differentially expressed circRNAs (44 downregulated and 50 upregulated) in the peripheral blood cells of 20 patients compared to 20 unaffected controls [253] However, direct comparisons between these studies are limited, as the latter study did not include a summary table detailing the differential expression of circRNAs and their corresponding host gene annotations. A meta-analysis integrating these datasets could provide a more robust understanding of circRNA dysregulation in bipolar disorder.

Several studies have reported differences in circRNA expression profiles in the prefrontal cortex regions of bipolar disorder patients, including DLPFC, OFC [223], anterior cingulate cortex (ACC) [254] and medial frontal gyrus (MFG) [255]. These regions play critical roles in cognitive, emotional, and executive functions. Even subtle dysfunctions in these interconnected neuronal networks can disrupt their coordinated activity, contributing to the development of mental health disorders. Comparing these studies, 45 genes produce circRNAs dysregulated in at least two brain regions. Interestingly, one gene, *NALCN* (*sodium leak channel, non-selective*), was found to express a circRNA dysregulated in different brain regions and cohorts, albeit with discordant direction (downregulated in OFC [223] and upregulated in MFG [255]). NALCN is a voltage-gated ion channel responsible for the regulation of Na+ permeability to control neuronal excitability [256]. However, the functional role of circNALCN in the nervous system and its association with BD remains unknown.

Of particular interest is a circRNA derived from the *HOMER1* gene, circHOMER1, which is consistently downregulated in the DLPFC and OFC of BD patients [223]. Genetic variation in the *HOMER1* locus near a distal enhancer region (rs6865469, *p* = 1.65e–08) is significantly associated with bipolar disorder [252]. How this variation in the *HOMER1* regulatory region affects circRNA biogenesis remains unclear. Human stem cell-based assays with CRISPR knock-in and knockout approaches to generate stem cell lines with different allelic combinations could help elucidate the effect of these genotypes on circRNA expression. CircHOMER1 is also reduced in iPSC-derived neuronal cultures from BD patients. In vivo knockdown of circHOMER1 in the OFC of mice alters the expression of the HOMER1B mRNA isoform and numerous alternative transcripts from genes involved in synaptic plasticity and psychiatric disorders [223].

Bipolar disorder patients often struggle with adapting their behavior to changing circumstances, a deficit perceived as impaired cognitive flexibility. In mice, reduced circHOMER1 expression in the OFC impairs behavioral flexibility, as demonstrated in a reversal learning test [150]. The mechanism of action for circHOMER1 in both schizophrenia and bipolar disorder appears to involve its direct interaction with the 3′ UTR of HOMER1B mRNA and the sequestration of ELAVL4, an RBP required for HOMER1B expression in the synapses (Fig. 3C). The regulation of RNA processing into linear and circular RNA isoforms depends on the profile of RBPs expressed in each brain regions and cell type, as well as the genetic variations associated with each disorder, potentially underlying the disease-specific patterns of coding and non-coding RNA signatures.

Another study identified circCCNT2 upregulated in the ACC of 13 BP patients when compared to 13 neurotypical controls, a finding replicated in an independent cohort of 24 patients and 27 controls [254]. The *CCNT2* (cyclin T2) gene encodes a cyclin protein that regulates CDK9 (cyclin dependent kinase 9) activity, promoting the phosphorylation of RNA polymerase II and other transcription factors [257]. Bioinformatic predictions suggest that circCCNT2 may interact with over 25 RBPs and potentially serve as a sponge for miR-877-5p, which is predicted to target genes involved in synapse formation. However, these hypotheses require experimental validation. Interestingly, circCCNT2 expression is reduced in B lymphoblastoid cells from patients following lithium treatment, whereas no change is observed in unaffected controls [257]. The authors propose circCCNT2 as a potential alternative treatment target for patients who experience adverse effects or do not respond to lithium therapy [254]. Although preliminary, these findings provide compelling evidence for circCCNT2’s role in BD and its potential utility in developing novel therapeutic approaches

### Major depressive disorder

Approximately one in five individuals will be affected by major depressive disorder (MDD) during their lifetime [258, 259]. MDD has a heritable component, with up to 37% of the risk explained by genetic variation across at least 44 risk loci [260], however it is more likely to result from a combination of environmental and genetic factors. High comorbidity with other psychiatric disorders as well as differences in personality, sex, and age, also contribute to the risk of developing MDD [261]. Due to the intricate interplay between genes, environmental factors, and the limited understanding of the underlying molecular mechanisms, treatment for MDD is often suboptimal. This underscores the need to explore all potential avenues, including the contribution of both protein-coding and non-coding genes, to identify gene networks and molecular mechanisms associated with the disorder. While some studies suggest that circRNAs may play a role in MDD, the evidence remains limited, though some investigations have started to uncover their potential involvement in the disease pathology [220, 221, 262–265].

A recent study suggests that a circular RNA derived from exons 4, 5 and 6 of the *DYM* (*dymeclin*) gene (circDYM) could serve as a novel therapeutic target for MDD [266]. CircDYM was found to be downregulated in the peripheral blood of MDD patients, as well as in the plasma and hippocampus of two depressive-like mouse models. Overexpression of circDYM in mice ameliorated depressive-like symptoms, indicating its potential therapeutic relevance. The study reveals that circDYM functions as a sponge for miR-9, regulating downstream miR-9 target genes, including *HECTD1* (HECT domain E3 ubiquitin protein ligase). Reduced circDYM expression leads to increased bioavailability of miR-9, resulting in the downregulation of *HECTD1*. This, in turn, reduces HSP90 ubiquitination, which promotes microglial activation and enhances neuroinflammation. Further supporting these findings, previous studies have demonstrated increased miR-9 expression in the nucleus accumbens and striatum of a depressive-like mouse model [267, 268]. Additionally, neurons exporting miR-9-containing exosomes that promote M1 polarization in microglia, leads to the release of proinflammatory cytokines [269]. These findings highlight a potential mechanism by which circDYM downregulation exacerbates neuroinflammation by increasing miR-9 activity, leading to dysregulation of microglial function (Fig. 3D).

Recent investigations have revealed changes in circRNA expression in depressive-like animal models following treatment with antidepressant compounds, including traditional Chinese medicine [270] and plant-derived therapies [262]. One study demonstrated alterations in circRNA expression profiles in a depressive-like rat model treated with Xiaoyaosan (XYS), a traditional Chinese medicine formula with known antidepressant effects. Rats exhibiting depression-like behaviors and treated with XYS showed differential expression of 28 circRNAs [270]. XYS treatment improved depression symptoms by increasing locomotor activity and sucrose preference while reducing immobility time during forced swimming tests. Additionally, XYS attenuated synaptic loss in the hippocampus, potentially through modulation of the PI3K/Akt signaling pathways, a well-established target of conventional antidepressants. However, the identities of affected circRNAs and the precise mechanisms by which XYS influences their expression remain largely unknown [270].

In another study, a depressive-like mouse model was treated with geniposide (GP), a plant-derived compound. GP restored the expression of circ_0008405 (homolog of circPBX1), a circRNA that was downregulated in depressive-like mice, leading to amelioration of depression-like symptoms. Circ_0008405 acts as a miRNA sponge for miR-25-3p, increasing the expression of its target genes, including *Gata2* [262]. Interestingly, a previous study found that overexpression of human *GATA1* and *GATA2* induced depressive behavior in rats [271]. These findings suggest that dysregulation of circRNA expression, such as the reduction of circ_0008405, contributes to depressive-like behaviors. Treatment with GP not only normalized circRNA expression but also ameliorated depression-like symptoms, highlighting the therapeutic potential of targeting circRNA networks to treat depression.

### Alzheimer’s disease

Alzheimer’s disease (AD) is a progressive neurodegenerative disorder characterized by the aggregation of hyperphosphorylated tau protein into intracellular neurofibrillary tangles and amyloid beta (Aβ) peptide into extracellular plaques. These pathological changes lead to memory loss, cognitive decline, and impairment in executive functions [272, 273]. AD is a complex multifactorial condition with a broad spectrum of manifestations, ranging from early-onset cases – frequently associated with rare autosomal dominant mutations in *APP* (*amyloid beta precursor protein*), *PSEN1* (*presenilin 1*) and *PSEN2* (*presenilin 2*) genes – to late-onset sporadic cases, which are associated with common alleles of small effect sizes that in aggregation contribute to genetic susceptibility to AD [274, 275]. Dysregulated ncRNAs, including circRNAs, have been implicated in the regulation of amyloid plaque formation and progression, playing critical roles in AD pathophysiology [276, 277]. These findings suggest that circRNAs may influence key molecular pathways underlying AD, providing opportunities for therapeutic intervention. Numerous studies have also highlighted the role of circRNAs in the etiology and severity of AD [126, 278–281], and some circRNAs have been proposed as potential biomarkers for diagnosing and monitoring the progression of AD in studies using peripheral blood cells, plasma, and cerebrospinal fluid [282–286].

A meta-analysis has provided an atlas of circRNA expression changes in cortical regions of AD patients, revealing significant correlations with clinical and neuropathological traits of AD [278]. This analysis identified 164 circRNAs dysregulated in the brains of AD patients, several of which are co-expressed with AD-associated genes involved in brain hypometabolism and clinical traits. In particular, circHOMER1 is significantly downregulated in AD patients and strongly correlates with disease severity as indicated by the clinical dementia rating and Braak score – a measure of AD severity based on distribution and density of neurofibrillary tau tangles in the brain. Furthermore, circHOMER1 contains multiple putative binding sites for miR-651, a miRNA predicted to target key AD-associated genes, including *PSEN1* and *PSEN2* [278]. Reduced circHOMER1 expression increases miR-651 bioavailability, enhancing miRNA-mediated suppression of PSEN1 and PSEN2, potentially impairing γ-secretase activity, which is essential for amyloid beta processing (Fig. 3E). In contrast, circCORO1C is upregulated in AD patients and associated with clinical traits and AD-associated genes including *APP*. Acting as a sponge for miR-105, circCORO1C reduces miR-105 bioavailability, leading to increased *APP* expression, which may exacerbate amyloid plaque formation and synaptic dysfunction (Fig. 3F). These findings are supported by independent studies and illustrate the potential regulatory roles of circHOMER1 [287–289] and circCORO1C [283, 288] in AD pathogenesis.

A circRNA derived from the APP gene (circAβ) has been identified in the prefrontal cortex of AD patients [126]. CircAβ encodes a novel 175-amino acid amyloid beta polypeptide, which can be processed into amyloid beta peptides, suggesting an alternative pathway for amyloid beta biogenesis (Fig. 3G). Another circRNA, circPSEN1, is upregulated in AD patients [290] and is proposed to act as a sponge for miR-4668-5p and miR-5584-5p. These miRNAs regulate genes involved in TGF-β1 and Notch signaling pathways, which are critical to AD progression [291–293]. Interestingly, autosomal dominate AD studies have revealed circPSEN1 isoform dysregulation without changes in PSEN1 mRNA expression [294].

Overexpression of circRIMS2 in the hippocampus of an AD mouse model caused a reduction in dendritic spine density and memory deficits [281]. Amyloid β was found to enhance METTL3-dependent m6A modification of circRIMS2, increasing its stability and promoting more efficient sponging of miR-3968. Mir-3968 is known to inhibit *UBE2K (ubiquitin conjugating enzyme E2 K*) expression, which can alter synaptic transmission via ubiquitination of GRIN2B (glutamate ionotropic receptor NMDA type subunit 2B) protein. Increased circRIMS2 expression resulted in more UBE2K activity, leading to increased ubiquitination and degradation of GRIN2B protein, causing synaptic dysfunction [281, 295, 296]. Co-overexpression of miR-3968 and circRIMS2 in mouse model restored dendritic spine density and memory performance to healthy-like levels. Interestingly, RIMS2 mRNA levels were consistently downregulated in the hippocampus of AD patients across multiple independent studies [297–299], suggesting AD-specific splicing disruptions at the *RIMS2* locus [300]. Aberrant splicing, possibly caused by a 5′ splice site variant, has been implicated in exon skipping and nonsense-mediated mRNA decay [301]. These findings highlight the multifaceted impact of circRIMS2 dysregulation on synaptic function and its potential role in AD pathophysiology.

### Parkinson’s disease

Parkinsons disease (PD) is the second most common neurodegenerative disorder globally, with its prevalence increasing significantly with age. The disease manifests with a combination of motor symptoms, including tremors, bradykinesia, and rigidity, and non-motor symptoms, such as cognitive impairment, sleep disturbances, and autonomic dysfunction. PD is typically characterized by the progressive loss of dopaminergic neurons in the substantia nigra and the accumulation of protein aggregates containing α-synuclein [302, 303]. A large-scale multi-ancestry meta-analysis of GWASs identified 78 independent risk loci associated with PD, with the majority of genetic variations (95%) occurring in regulatory non-coding regions [304]. Notably, this includes variations in genes expressing circRNAs, such as the *α-synuclein* (*SNCA*) gene, which plays a central role in PD pathogenesis. Emerging evidence suggests that circRNAs may serve as biomarkers for diagnosis and assessment of PD severity [305–310]. Furthermore, other studies have investigated the involvement of circRNAs in key pathogenic mechanisms, including their regulation of α-synuclein aggregation, neuroinflammation, apoptosis, autophagy, and mitochondrial dysfunction, with much of this research conducted using animal and cell models of PD [27, 311–321].

A study involving 300 PD patients and 100 healthy controls identified circulating cell-free circRNA as potential plasma biomarkers for PD [322]. Two diagnostic panels were developed: one consisting of two circRNAs (circARID1B and circTCONS_l2_00002816) demonstrated high sensitivity and specificity for early diagnosis of PD, while a second panel, comprising four circRNAs (circFAM83H, circHUWE1, circARID1B and circTCONS_l2_00002816), was able to differentiate late-stage from early-stage PD [322]. Another novel biomarker and potential therapeutic target for PD, circEps15, was recently identified in both human and animal studies [317]. This circRNA was found to be downregulated in the plasma of PD patients and significantly correlated with disease progression. Consistent with patient findings, reduced circEps15 expression was observed in plasma and midbrain samples from a chemically induced mouse model of PD. Overexpression of circEps15 in these mice and SH-SY5Y cells was found to promote dopaminergic recovery in vitro through improved mitochondrial function. Mechanistically, circEPS15 functions as a miR-24 sponge promoting stable expression of target gene *PINK1* thus enhancing PINK1-PRKN-dependent mitophagy to eliminate damaged mitochondria and maintain mitochondrial homeostasis in neurons [317].

A study reveals that circRNAs tend to accumulate in an age-dependent manner in several brain regions in healthy individuals but in PD patients this correlation is lost in substantia nigra where total number of circRNAs is reduced [313]. In contrast, the same study reports that circSLC8A1 increases in the substantia nigra of PD patients and in cultured cells exposed to oxidative stress induced by Paraquat. CircSLC8A1 carries several binding sites for miR-128 and identified to interact with AGO2. This strongly suggests this circRNA functions as a sponge affecting the expression of miR-128 target genes. However, the exact functional impact caused by miR-128 sponging remains unknown.

Other studies in animal and cell line models identified several dysregulated circRNAs, such as circSNCA [311], circPANK1 [316], circHIPK3 [319], circHIVEP2 [321], circDLGAP4 [314] and circSAMD4A [315]. All these circRNA are proposed to function as miRNA sponges affecting biological pathways associated with PD such as α-synuclein aggregation, neuroinflammation and degeneration, and mitochondrial dysfunction. Notably, circPANK1 and circSNCA are proposed to function as miR-7 sponges and found upregulated in PD mouse and cell models. One such cell model using SH-SY5Y cells treated with a neurotoxin (1-Methyl-4-phenylpyridinium) found increased circSNCA expression [311]. CircPANK1 was found to be upregulated in the substantia nigra of a PD mouse model treated with rotenone, another compound used to mimic key pathological traits of disease [316]. Increased expression of these two circRNAs upregulates α-synuclein SNCA protein expression by reducing miR-7 bioavailability and enhancing the expression of target genes, such as *SNCA*, thus increasing risk of SNCA aggregation and neurodegeneration of dopaminergic neurons [311, 316] (Fig. 3H).

## Potential clinical applications of circRNAs

The stability, specificity, and abundance of circRNAs render them as not only potentially diagnostic tools but also as novel therapeutic targets. The aberrant expression and resulting dysregulation in normal functioning of circRNAs in neurological conditions is becoming a more prominent area of research, with fascinating discoveries being made. In the last two decades, new insights have been made into circRNA and their interplay with regulatory mechanisms that underpin complex and typically highly polygenic neurological conditions such as schizophrenia, bipolar disorder, depression and as well as neurodegenerative conditions like Alzheimer’s disease and Parkinsons disease. One of the most compelling aspects of circRNAs for studying neurological conditions lies in their abundant expression in the brain [29, 59], where they are highly expressed in neuronal cells comparatively to other cell types, as well as other cell-type specific expression patterns [25]. CircRNAs are also highly stable, have unique transcript sections across backsplice junction region, and tend to have low immunogenicity, all of which are features that can aid in developing more robust RNA based therapeutics [13, 209, 233].

As detecting circRNAs has become more streamlined, plasma derived circRNAs have also shown promise for developing diagnostic biomarkers and panels dedicated for early detection and monitoring of neurological conditions [221, 322–324]. The versatility of circRNAs as biomarkers in neurological conditions is underscored by their presence in cerebrospinal fluid, offering a valuable diagnostic window into central nervous system milieu [324, 325]. New possibilities for leveraging circRNAs as potential biomarkers in neurological conditions are also being determined in urine and saliva [265, 326], presenting additional non-invasive avenues for biomarker discovery in neurological conditions. There has also been some research into circRNA nanoparticle delivery platforms, viral and lipid based, with limited success as the immense complexity of the brain not just in structure but also function has proven quite challenging [327–330]. Additionally, synthetically produced circRNAs aptamers could also be used as a therapeutic themselves to help modulate conditions associated with protein dysfunction such as neurodegenerative conditions [331].

Given that some circRNAs present regulatory patterns enabling their translation into protein sequences [124, 128–132, 332], a comprehensive understanding of the mechanisms underlying their translational capability could facilitate the development of more stable and effective RNA-based therapies. CircRNAs offer advantages over mRNA treatments, including enhanced stability, prolonged translation duration, and reduced immunogenicity. Recently, circRNA-based vaccines have been proposed as innovative strategies for achieving durable and effective expression of viral and cancer antigenic proteins. Examples include circRNAs encoding the SARS-CoV-2 spike protein [333] and charge-altering releasable transporter (CART)-encapsulated circRNAs encoding antigens against targeting cancers [334].

## Challenges and future directions

Research into circRNAs is still in early stages, particularly regarding their clinical applications in neurological conditions. The high stability of circRNAs compared to mRNA and their cell-specific expression profiles in neurodevelopment and normal brain function make them promising candidates for diagnostics and therapeutics. Using circRNAs as biomarkers for diagnostics may be attainable with machine learning methods and larger sample sizes profiling various biofluids and cellular biopsies, such as serum, saliva, cerebrospinal fluid, and patient-derived cells and tissue cultures. However, the precise mechanisms and impact of circRNA functions in brain homeostasis, as well as their contributions to the etiology of neuropsychiatric and neurodegenerative conditions, require further investigation before their clinical application in therapies can be realized. Moreover, targeting specific brain regions for treatment poses significant challenges, such as overcoming the blood-brain barrier and developing cost-effective, targeted delivery systems for circRNA.

RNA sequencing remains the predominant method for circRNA identification; however, a lack of standardized protocols and detection tools limits precision and specificity [169]. The absence of standardized nomenclature for circRNAs further complicates research communication and replication. Databases such as circBank [153] have incorporated conversion tools to address this issue but incomplete reporting of circRNA transcript lengths, often due to short-read sequencing limitations, persists as a significant challenge. Long-read sequencing technologies, coupled with advanced bioinformatic tools like CIRI-long [176], are beginning to overcome these limitations, enabling precise determination of circRNA structures, interaction sites, and functional roles.

Developing effective and standardized diagnostic biomarkers for neurological conditions has been notoriously difficult. Despite promising studies identifying circRNAs as potential biomarkers, most findings are based on small sample sizes and limited datasets. The intricate nature of gene expression profiles in the brain and the lack of standardized protocols poses a major hurdle in clinical applications [270, 288, 325, 335]. Larger-scale cohorts and more robust experimental models are required to develop reliable diagnostic panels. Patient-derived neuronal cultures, brain organoids, and animal models offer powerful tools for characterizing circRNA functions and evaluating whether circRNA biogenesis is globally impaired or restricted to specific cell types and brain regions. These models can help elucidate how circRNA dysregulation contributes to clinical features of various neurological disorders.

Importantly, some circRNAs have demonstrated potential for early diagnosis or for predicting disease severity [234, 322, 336]. However, the diversity in patient populations and the cell-specific regulation of circRNA expression require stringent standardization of sample types, biofluids, and cell sources. Combining circRNA biomarkers with other molecular modalities, such as miRNAs and mRNAs could lead to the development of effective diagnostic panels. Ongoing research is uncovering pathways related to circRNA function and biogenesis, highlighting their potential as biomarkers and therapeutic targets. A recent study demonstrated the biological relevance of circRNAs by creating a circRNA-deficient mouse model in which the splice acceptor site for circTulp4 was specifically mutated without affecting the expression of Tulp4 mRNA or protein [83]. This study elegantly demonstrates that circTulp4 plays a critical role in excitatory neurotransmission and sensitivity to aversive stimuli, showing the importance of circRNAs in regulating neuronal functions.

While this study demonstrated that certain regulatory patterns can be modulated to disrupt circRNA expression without affecting the host gene mRNA, canonical splice acceptor-donor sites cannot always be altered without interfering with mRNA splicing. An alternative approach could involve a more detailed investigation into circRNA-specific motif pattern associated with RBPs and co-factors that are directly linked to circRNA backsplicing mechanisms. Understanding these molecular interactions and the precise mechanisms controlling when and how circRNAs are processed could pave the way for novel experimental strategies. These approaches would enable the functional role of circRNAs to be specifically validated, independently of their host gene mRNA, offering deeper insights into their distinct regulatory capabilities.

One significant hurdle to RNA-based therapies, including those involving circRNAs, is the lack of effective delivery systems for synthetic RNAs targeting specific brain cells. Moreover, the stability and toxicity of RNA sequences in human organs and tissues, including the brain, will require substantial advancements in medicinal chemistry before safely moving forward into clinical trials. Promising progress is being made in delivery mechanisms, including nasal spray formulations, ultrasound-based techniques, and vasoactive agents, which may help overcome the blood-brain barrier [191, 337]. Furthermore, the hydrophilic and negatively charged nature of RNA molecules poses several challenges for cellular uptake, emphasizing the need for innovative delivery technologies.

Due to their ability to encode functional peptides, superior stability, and extended lifespan compared to mRNAs, circRNAs offer a promising platform for advancing RNA therapeutics. They hold potential as a powerful tool for pharmaceutical peptide production and gene therapy applications. By overcoming existing challenges related to delivery, stability, and characterization, circRNAs could unlock significant opportunities for individualized therapies of neurological disorders. Translating circRNA research into practical clinical applications could drive the development of innovative diagnostics and therapeutics, addressing critical gaps in the personalized treatment of neurological conditions.

## Conclusion

Significant advancements have been made in uncovering the functional roles, biogenesis, and molecular mechanisms of circRNAs in neurological dysfunction. These advancements include neuropsychiatric conditions such as schizophrenia, bipolar disorder, and major depressive disorder, as well as neurodegenerative conditions like Alzheimer’s and Parkinson’s disease. Insights into the spatio-temporal expression and functional dynamics of circRNAs in these conditions are fostering the development of potential clinical applications. The findings discussed in this review illustrate the intricate regulatory roles circRNAs play in neurological conditions, particularly through interactions with miRNAs and RBPs. Select circRNAs have demonstrated functional effects in model organisms, elucidating their involvement in key neurological pathways. The polygenic nature of these disorders, coupled with their interplay with environmental factors, underscores the need for more advanced model systems. Patient-derived stem cells and differentiated neuronal tissues also offer a promising avenue for identifying novel mechanisms and therapeutic targets, providing valuable insights into human-specific phenotypes. Thus, the role of circRNAs, as non-coding regulatory molecules, represents a transformative shift in our understanding of neurological disorders. Their unique properties and regulatory roles hold promise for the development of more effective diagnostic tools, biomarkers, and therapeutic interventions. This evolving field offers new hope in addressing the complexities of neurological disorders, potentially paving the way for innovative, personalized approaches to treatment and management.
